# A Simulation Analysis and Screening of Deleterious Nonsynonymous Single Nucleotide Polymorphisms (nsSNPs) in Sheep *LEP* Gene

**DOI:** 10.1155/2022/7736485

**Published:** 2022-08-08

**Authors:** Shishay Girmay, Hafiz Ishfaq Ahmad, Quratul Ain Zahra

**Affiliations:** ^1^Department of Animal Science, College of Dryland Agriculture, Samara University, Ethiopia; ^2^Department of Animal Breeding and Genetics, University of Veterinary and Animal Sciences, Ravi Campus, Pattoki, Pakistan; ^3^Woman Medical Officer DHQ Hospital Rajanpur, Punjab, Pakistan

## Abstract

Leptin is a polypeptide hormone produced in the adipose tissue and governs many processes in the body. Recently, polymorphisms in the LEP gene revealed a significant change in body weight regulation, energy balance, food intake, and reproductive hormone secretion. This study considers its crucial role in the regulation of the economically important traits of sheep. Several computational tools, including SIFT, Predict SNP2, SNAP2, and PROVEAN, have been used to screen out the deleterious nsSNPs. Following the screening of 11 nsSNPs in the sheep genome, 5 nsSNPs, T86M (C → T), D98N (G → A), N136T (A → C), R142Q (G → A), and P157Q (C → A), were predicted to have a significant deleterious effect on the LEP protein function, leading to phenotypic difference. The analysis of proteins' stability change due to amino acid substitution using the I-stable, SDM, and DynaMut consistently confirmed that three nsSNPs (T86M (C → T), D98N (G → A), and P157Q (C → A)) increased protein stability. It is suggested that these three nsSNPs may enhance the evolvability of *LEP* protein, which is vital for the evolutionary adaptation of sheep. Our findings demonstrate that the five nsSNPs reported in this study might be responsible for sheep's structural and functional modifications of LEP protein. This is the first comprehensive report on the sheep *LEP* gene. It narrow downs the candidate nsSNPs for *in vitro* experiments to facilitate the development of reliable molecular markers for associated traits.

## 1. Introductions

Leptin is one of the major hormones secreted by adipocytes. It is the primary function of regulating homeostatic control of energy balance, metabolism, neuroendocrine system, and other functions through its effects on the central nervous system [1, 2].

The short negative feedback maintains the body leptin proportion through pituitary cells, or *via* the long feedback performed by neurosecretory cells, interacting with corresponding receptors in the hypothalamus (paraventricular, lateral, ventromedial, and dorsomedial nuclei), leads to repression of orexigenic peptide production and stimulation of anorexigenic factors [3]. Then, the brain regulates the balance of energy expenditure from the body and the amount of energy stored in the body of the organism [4]. Usually, blood leptin levels are proportionate to the mass of adipose tissue, so the fatter individuals have, the more leptin they have circulating in their blood. Nonetheless, people and animals with a higher level of adipose tissue seldom show leptin synthesis or leptin production [5]. The reduction in the hormone's effectiveness may be associated with a fault in drawing hormonal signals or the ability of leptin to penetrate the blood-brain barrier, resulting in leptin resistance [6]. In turn, leptin deficiency or polymorphisms in the leptin pathways increase appetite and energy intake, ultimately leading to obesity [7].

Single nucleotide polymorphisms are essential and the most common types of mutations associated with complex traits [8]. In particular, the missense mutations located in the coding region, which alter the amino acid configuration, may cause a significant change in the structure and function of the native protein [9]. *In vitro*, functional comparison of mutated proteins with their corresponding wild-type proteins associated with specific traits of animals is a common practice that enables us to identify the impact of each nsSNP in the corresponding protein [10, 11]. However, the experimental design for each mutational change is laborious and time-consuming. Therefore, it is reasonable and economical to carry out the basic work required for mutation data mining and predict its effect on protein properties through computational analysis (Nailwal and Chauhan, 2017). Screening out the deleterious nsSNPs with a significant consequence on phenotype from the tolerant nsSNPs (without phenotypic changes) has a pivotal role in understanding the molecular basis of the polygenetic traits in sheep. Therefore, considering the role played by the *LEP* gene in regulating body weight, energy balance, and feed intake traits in sheep, we retrieved all of the mutations found in sheep *LEP* gene deposited in the Ensembl sheep genome browser until March 2022. The objective of this study was to characterize the potential variations in the ovine LEP gene using *in silico* analysis.

## 2. Materials and Methods

**Figure 1:**
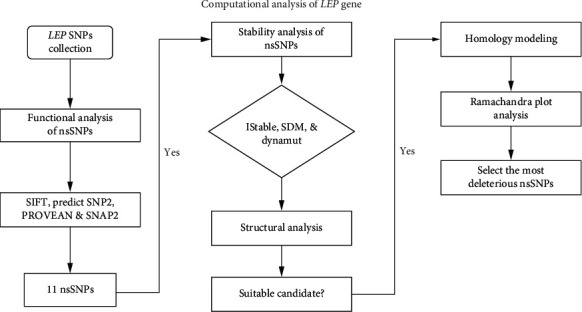
Diagrammatic representation of computational tools used for the analysis of *LEP* gene.

We used various computational tools to screen out the functional effects of the nsSNPs in the *LEP* gene. Details of each bioinformatics tool are presented below and summarized in Figure 1.

### 2.1. Collecting SNPs and Protein's Sequence from the Databases

Sheep leptin (*LEP*) gene SNPs were collected from the *Ovis aries* Ensembl genome browser 106 (https://uswest.ensembl.org/Ovis_aries/Info/Index) [12]. The transcript sequence and the protein encoded by the sheep *LEP* gene were retrieved from the Ensemble database (https://ensembl.org/) [13]. Then, the UniProt ID for the amino acid (UniProtKB - W5NWK7) was obtained from UniProt Protein Database (http://www.uniprot.org).

### 2.2. Nonsynonymous SNP Functional Analysis for *LEP*

Four tools, SIFT, PredictSNP2, PROVEAN, and SNAP2, were used to predict the practical context of missense mutations in the *LEP* gene.

Firstly, the amino acid substitutions that affect protein structure and function lead to phenotypic changes were screened by SIFT (Sorting Intolerant From Tolerant; http://sift.jcvi.org/) tool [14]. The identification numbers (rsIDs) of each nsSNP of the *LEP* gene obtained from Ensembl were submitted as a query to SIFT for homology searching. Results were obtained as SIFT scores which were classified as damaging (0.00–0.05), potentially damaging (0.051–0.10), borderline (0.101–0.20), or tolerant (0.201–1.00) [15]. Then, PredictSNP2 (http://loschmidt.chemi.muni.cz/predictsnp2) was used to identify the deleterious nsSNPs. PredictSNP2 is a consensus classifier for predicting the effect of amino acid substitutions based on the output of eight different amino acid-based predictors http://loschmidt.chemi.muni.cz/predictsnp [16].

PROVEAN (Protein Variation Effect Analyzer: http://provean.jcvi.org/index.php) is used to predict the level of impact on protein structure and biological function. A protein FASTA sequence with amino acid alterations is used as the input query. It classifies nsSNPs as deleterious or neutral based on whether the final score falls below a threshold of − 2.5. Scores over this threshold are considered neutral [17].

SNAP2 predicts the functional impact of mutations [18]. SNAP2 predicts the functional impact of mutations (Turkson, 2004). SNAP2 is a taught classifier based on the “neural network” machine learning device. It uses a number of sequence and variant properties to discriminate between effect and neutral variants/nonsynonymous SNPs. The SNAP2 achieved sustained two-state accuracy (effect/neutral) of 82 percent in cross-validation across 100,000 experimentally annotated variations (at an AUC of 0.9). In other words, this represents a major improvement over existing methods [19] (the website https://rostlab.org/services/snap2web/ is where you may find it).

### 2.3. Analysis of Protein's Stability Change upon Amino Acid Substitution

Protein evolution is mainly governed by protein stability [20]. After folding, we analyzed the relationship between LEP mutations and protein stability based on a smaller free energy change (*Δ*G or dG). In contrast, the difference in folding free energy change between wild-type and mutant protein (*ΔΔ*G or ddG) is often considered an impact factor on protein stability changes [21]. The protein structure stability and the molecular and structural effects of protein-coding variants were predicted using the integrated predictor for protein stability change upon a single mutation called iStable (http://predictor.nchu.edu.tw/iStable) [22]. Furthermore, the change in protein stability upon mutation was estimated using a site-directed mutator (SDM) (http://marid.bioc.cam.ac.uk/sdm2/prediction) a server [23]. Finally, the DynaMut (http://biosig.unimelb.edu.au/dynamut/) was used to verify the impact of mutations on protein conformation, flexibility, and stability and to visualize the protein dynamics [24].

### 2.4. Structural Conformation and Conservation Analysis of *LEP*

Highly conserved functional regions of the protein coded by the *LEP* gene was identified by ConSurf tool (http://consurf.tau.ac.il/) [25–27].

### 2.5. Prediction of Secondary Structure

The secondary structure of *LEP* was predicted using the PSIPRED server available at (http://bioinf.cs.ucl.ac.uk/psipred/) [28]. It is based on a two-stage neural network with the implementation of position-specific scoring matrices constructed from PSI-BLAST to predict the available secondary structures of a protein [29].

### 2.6. Homology Modelling

The 3D structures of the protein encoded by LEP were constructed using four different homology modeling tools. No crystal structure with an appropriate length of this protein was available in the protein data bank.

#### 2.6.1. Homology Modelling by Swiss-Model Server

Swiss-Model workplace available at (https://swissmodel.expasy.org/), a web-based tool for the homology modeling of protein, was used to avail the three-dimensional structure of LEP [30, 31]. The CAMEO system calculates the accuracy of the constructed model. Swiss-Model is an automated tool based on evolutionary information which searches for the best sequence-template alignment from its high-throughput template library (SMTL) to build the model [32].

#### 2.6.2. Homology Modelling by Phyre-2 Server

Phyre-2 tool (http://www.sbg.bio.ic.ac.uk/phyre2/), based on the Hidden Markov method, was used to predict the homology-based three-dimensional structure of the query amino acid sequence. It combines five steps to build a model: (1) collecting homologous sequence, (2) screening of fold library, (3) loop modelling, (4) multiple template modeling by ab initio folding simulation Poing, and (5) side chain placement [33, 34].

#### 2.6.3. Homology Modeling by RaptorX Server

RaptorX server available at (http://raptorx.uchicago.edu/StructurePrediction/predict/), using RaptorX-Boost and RaptorX-MSA to construct the three-dimensional structure of a protein, predicted the 3D model of the protein coded by CDKN1A. It combines a nonlinear scoring function and a probabilistic consistency algorithm to predict the model structure [35].

#### 2.6.4. Verification of Three-Dimensional Model of Protein

RAMPAGE Ramachandran plot analysis was used for the verification of 3D structures. It provides the number of residues in the favored, allowed, and outlier regions [36]. If a good proportion of residues lie in the favored and allowed region, then the model is predicted to be good.

## 3. Results

### 3.1. Data Mining

Data mining of the sheep genome browser from Ensembl database on March 12, 2022, revealed 543 SNPs of the *LEP* gene in sheep, of which 11 were missense mutations. Detailed information about these SNPs is shown in Table 1.

### 3.2. Nonsynonymous SNPs Functional Analysis for LEP

Out of the 11 nsSNPs subjected to SIFT tool to predict their impact level on the LEP protein function as deleterious or tolerated, the nine nsSNPs have shown a damaging effect with an average prediction score of 0.00–0.02, and the remaining two nsSNPs were tolerant (T86M and R196Q) Table 2.

PredictSNP2 further evaluated all the 11nsSNPs, including the nine predicted as deleterious by SIFT. The PredictSNP2 is a consensus classifier tool for predicting the effect of amino acid substitutions based on the output of eight computational prediction tools. The PredictSNP2 tool prediction outcomes revealed that eight nsSNPs were predicted as deleterious (Table S1). The SNAP2 was used to confirm further the sequence variants' anticipated effects on the LEP protein function.

### 3.3. Mutant Protein Stability Prediction for LEP

The iStable Meta server tool demonstrated that the amino acid changes in R2C, L35P, D98N, N136T, R142Q, P157Q, V181L, and R196Q cause loss and decreased stability of the LEP protein. However, the other three mutations (T75M, T86M, and V94I) increase the protein stability Table 3. These results were cross-checked with a site-directed mutator (SDM), and DynaMut servers found that eight nsSNPs significantly change the stability compared to the wild-type proteins.

Double-checking with site-directed mutator (SDM) and DynaMut servers consistently verified the molecular consequences of 5 nsSNPs. Out of these, three nsSNPs (T86M, D98N, and P157Q) increased stability by decreasing the molecular flexibility of the wild-type proteins. However, the other two nsSNPs (N136T and R142Q) revealed a decrease in stability and molecular flexibility Table S4.

**Figure 2:**
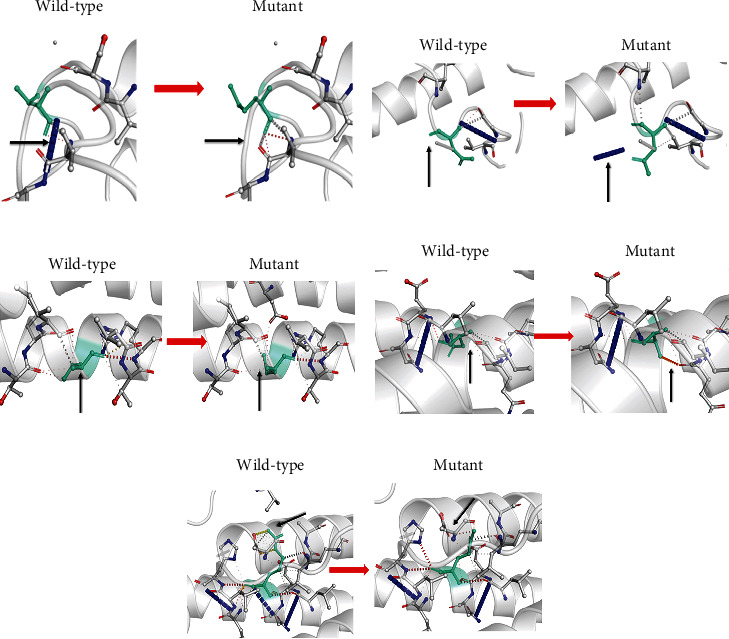
(a) (T86M): Change in the amino acid threonine into methionine at position 86. (b) (D98N): The amino acid aspartic acid change into asparagine at position 98. (c) (P157Q): Change the amino acid valine into isoleucine at position 157. (d) (N136T): Change the amino acid asparagine into threonine at position. (e) (R142Q): Change the amino acid arginine into glutamine at position 142.

Furthermore, the interatomic interactions prediction outcomes between wild type (left side) and after single point mutations (right side) are presented in Figures 2(a)–2(e). The wild-type and mutant residues are colored in light green and represented as sticks alongside the surrounding residues that take part in any interactions.

### 3.4. Structural Conformation and Conservation Analysis by ConSurf Server

Our ConSurf results indicated that nsSNPs at position L35P, T75M, V94I, D98N, and R142Q are in the highly conserved region, with a conservation score 9. Likewise, nsSNPs R2C, N136T, and P157Q have shown a conservation score of 8 (Figure S1). From this, we can speculate that these nsSNPs have a potential effect on LEP protein.

### 3.5. LEP Protein Secondary Structure Prediction by PSIPRED

The alpha helix and beta sheet distribution and the coil are exposed by PSIPRED (Figure S2). Among the secondary structures, the highest in percentage was coils (51.5%) followed by alpha helix (48.5%) and no beta-sheet (0.0%).

### 3.6. Homology Modelling

Structure refined and energy minimized homology models of LEP by Swiss-Model, Phyre-2, ConSurf, and RaptorX servers are illustrated using computational program QMEAN (Qualitative Model Energy ANalysis) (Figure S3). These models were checked for validation through Ramachandran plot analysis. Some residues in the model may lie in favorable (blue), allowed (green), or disallowed (red) regions of the Ramachandran plot. This coloring indicates residues that may have problems with the backbone phi/psi angles.

### 3.7. Ramachandran Plot Analysis

Ramachandran plot is an x-y plot of phi/psi dihedral angles between NC-alpha and Calpha-C bonds to evaluate a protein's backbone conformation. The Ramachandran plot of the wild-type protein in the Swiss-Model showed 132 residues (93.6%) in the favored region, 8 residues in allowing region (5.7%), and one residue (0.7%) in the outer region (Figure S4A). The LEP hypothetical protein built by Phyre2 revealed 135 residues (95.7%) in favored region, 4 residues (2.8%) in allowing region, and 2 residues (1.4) in the outer part. Similarly, the Ramachandran plot of the wild LEP protein model constructed by the ConSurf server demonstrates 158 residues (95%) in the favored region, 4 residues (2.4%) in the allowing part, and 3 residues (1.8%) in the outer region. The last server we have been used for homology modelling was RaptorX. Here, the Ramachandran plot analysis of the model produced with this tool shows that the number of residues in the favored region was 52 (96.3%), the number of residues in allowed region 2 (3.7%), and the number of residues in outlier region was 0 (0.0%). Overall, the homology models evidenced the models of LEP protein were good. They can be used for further experiments and a better understanding of the protein's biological activity.

## 4. Discussion

Missense mutations in genes have a profound impact on protein function. Extensive computational analysis of the phenotypic features attributed to nsSNPs may reveal the susceptible variants by interrupting the original function [37, 38]. In humans, mutations of the LEP gene have been associated with obesity in different populations [39]. The sheep *LEP* gene, located on chromosome 4, encodes 204 amino acids annotated with 11 domains and is currently associated with 543 variations [12].

Leptin is an endocrine hormone member of the long-chain helical cytokine family. It has multiple effects on regulating food intake, energy expenditure, body weight, and immune responses [40]. Polymorphism in the leptin gene has been suggested to be connected to cattle carcass composition differences [38, 41, 42]. For example, a nucleotide switched from cytosine (C) to thymine (T) causes an amino acid change in the plasma leptin circulation and a higher 12^th^ rib fat and marbling score [41]. In addition, the T-allele in cattle has been reported to be associated with fatter carcasses, whereas the C-allele is associated with leaner carcasses [43].

Numerous research studies have reported polymorphisms in the sheep leptin (*LEP*) gene [44, 45] and its association with food intake [46], growth traits [47, 48], and carcass and mutton quality traits [49, 50]. Consequently, amino acid substitutions in the *LEP* gene have been recommended as predictors of relative differences among individuals for such economic traits [51].

In this study, following the screening of nsSNPs as deleterious or neutral upon their effect on protein function using the SIFT, predictSNP2, PROVEAN, and SNAP2, five nsSNPs T86M (C → T, rs593507294), D98N (G → A, rs426762318), N136T (A → C, rs429690456), R142Q (G → A, rs409584889), and P157Q (C → A, rs1093355763) were predicted to have a significant impact on the protein structure, stability, and function by the majority of the tools. As described [37] in the human CDKN1A gene and *ADIPOR2* gene by [52], and the *CSN3* gene in cattle by [53], dbSNP-based studies have been reported. Our study employed similar tools to predict the impact of the nsSNPs in the LEP gene and found important results.

The sequence of a protein determines the protein's physicochemical properties, such as protein structure, protein thermodynamic stability, the ability to interact with other molecules, and catalytic capacity [54, 55]. Thus, these properties, in turn, determine protein function [56].

Further analysis of these nsSNPs on protein functionality was predicted indirectly based on the effects exerted on protein stability. First, the I-stable server predicted that all the 11 nsSNPs impacted protein stability. Then, the report from I-stable was crosschecked using SDM and DynaMut. The stability analysis verified by SDM and DynaMut servers consistently narrowed to 5 nsSNPs. Three nsSNPs (T86M, D98N, and P157Q) had increased protein stability, whereas the other two nsSNPs (N136T and R142Q) showed a destabilizing effect on the wild-type proteins. DynaMut predicts the molecular consequences of the mutations in-depth through changes in protein dynamics and stability from vibrational entropy change [24]. The DynaMut-prediction outcomes of the *Δ* vibrational entropy energy between wild-type and mutants (*ΔΔ*SVib ENCoM) for the 3 nsSNPs were − 0.046 kcal.mol^−1^.K^−1^, − 0.056 kcal.mol^−1^.K^−1^, and − 0.027 kcal.mol^−1^.K^−1^, respectively. Our data suggest that the vibrational entropy change of protein upon mutation of the amino acid decreases the molecular flexibility of the protein. Therefore, the three nsSNPs that increased the protein stability may increase evolvability by allowing a protein to accept a wider range of beneficial mutations while still folding to its innate structure [57]. Our findings are compatible with [58], who reported that the marginal protein thermostability is a consequence of the mutation-selection balance. Similarly, [57, 59] explained that mutants derived from highly stable variants of a given protein are more likely to retain their fold and are consequently more likely to develop novel or improved protein functions.

In contrast to the other two nsSNPs, N136T (A → C, rs429690456) and R142Q (G → A, rs409584889) were evidenced by reduced stability. Table 3 suggests that these missense mutations might lead to new functions of the leptin gene in sheep. This finding is analogous to [21] stating that the evolution of new enzymatic specificities is accompanied by a loss of the protein's thermodynamic stability (DDG), implying a trade-off between acquiring new function and stability. Most likely, the mutations confer new functions that destabilize [57]. Thus, in this study, the mutations that cause a destabilizing effect on the thermodynamic stability of the protein through increasing the molecular flexibility may constrain the native evolution of leptin protein in sheep. Taken all together, the effect of the deleterious nsSNPs in the *LEP* gene may have a diverse impact on the performance of the sheep for the particular traits. The results from this study provide promising insight for future *in vitro* studies to specify the impact of these sorted nsSNPs on sheep performance.

## 5. Conclusion

A simulation-based study to detect the nsSNPs using many computational programs employed in this study has revealed 5 amino acid substitutions: T86M (C → T, rs593507294), D98N (G → A, rs426762318), N136T (A → C, rs429690456), R142Q (G → A, rs409584889), and P157Q (C → A, rs1093355763) were found to be functionally deleterious. These nsSNPs in the sheep *LEP* gene may contribute to the functional discrepancies compared to the wild LEP gene, which significantly regulates energy homeostasis, body weight control, and reproduction traits. Most of the variants identified in this study are located in the conserved domain of leptin. We also calculated the free energy changes for mutants and wild-type LEP proteins to evaluate their stability. Our data provide evidence for the functional role of the 5nsSNPs, which is helpful for further in vitro study to facilitate the development of reliable molecular markers for future practical application in sheep breeding.

## Figures and Tables

**Table 1:** SNPs information.

Location	Allele	cDNA position	Protein position	Amino acids	SNP ID	Consequences
4 : 92508292	C/T	4	2	R/C	R2C	Missense variant
4 : 92519830	T/C	104	35	L/P	L35P	Missense variant
4 : 92519950	C/T	224	75	T/M	T75M	Missense variant
4 : 92519983	C/T	257	86	T/M	T86M	Missense variant
4 : 92521847	G/A	280	94	V/I	V94I	Missense variant
4 : 92521859	G/A	292	98	D/N	D98N	Missense variant
4 : 92521974	A/C	407	136	N/T	N136T	Missense variant
4 : 92521992	G/A	425	142	R/Q	R142Q	Missense variant
4 : 92522037	C/A	470	157	P/Q	P157Q	Missense variant
4 : 92522108	G/T	541	181	V/L	V181L	Missense variant
4 : 92522154	G/A	587	196	R/Q	R196Q	Missense variant

**Table 2:** nsSNP analysis by SIFT.

Sr. no	NsSNPs	Amino acid change	Using homologs in the protein alignment	
			Prediction	Score
1	rs587813135	R2C	Damaging	0.00
2	rs414488761	L35P	Damaging	0.00
3	rs1086818376	T75M	Damaging	0.00
4	rs593507294	T86M	Tolerated	0.12
5	rs592349134	V94I	Damaging	0.00
6	rs426762318	D98N	Damaging	0.00
7	rs429690456	N136T	Damaging	0.01
8	rs409584889	R142Q	Damaging	0.00
9	rs1093355763	P157Q	Damaging	0.02
10	rs420693815	V181L	Damaging	0.00
11	rs428185456	R196Q	Tolerated	0.41

**Table 3:** Predicted effect of the single amino acid change on protein stability.

Position	I-Mutant 2.0		MUpro		I-stable	
	DDG	Stability	Conf. Score	Stability	Conf. Score	Stability
R2C	− 1.23	Decrease	− 1	Decrease	0.780005	Decrease
L35P	− 1.17	Decrease	− 0.21615	Decrease	0.768006	Decrease
T75M	− 0.34	Decrease	0.43239	Increase	0.757411	Increase
T86M	− 0.44	Decrease	1	Increase	0.732889	Increase
V94I	− 0.82	Null	0.34720	Increase	0.640778	Increase
D98N	− 1.21	Decrease	− 0.2594	Decrease	0.620153	Decrease
N136T	− 0.34	Decrease	− 0.54347	Decrease	0.802931	Decrease
R142Q	− 0.51	Decrease	− 1	Decrease	0.795042	Decrease
P157Q	− 1.47	Decrease	− 0.68383	Decrease	0.815634	Decrease
V181L	− 1.49	Decrease	0.229966	Decrease	0.526905	Decrease
R196Q	− 2.17	Decrease	Null	Null	0.651297	Decrease

DDG: predicted free energy change value (DDG), DDG < 0: decrease stability, DDG > 0.5 increase stability.

## Data Availability

All datasets generated/analyzed for this study are included in the manuscript and submitted as supplementary.

## Supplementary Materials

Supplementary Materials Supplementary Materials Table S1. Deleterious SNPs predicted by predictSNP2. Table S2. nsSNP analysis by SNAP^2^. Table S 3. nsSNP analysis by PROVEAN. Table S4. SDM and DynaMut tools analysis results of the effect of missense mutations on protein stability. Figure S1. Prediction of evolutionary conserved amino acid residues by ConSurf server. Conservation score is represented as the color coding bars. Figure S2. Protein secondary structure predictions by PSIPRED tool. The graphical output of PSIPRED prediction of secondary structure of the sheep LEP protein shows 6 *α*-helices extends from 41^th^ to 55^th^, 66^thto^ 84^th^, 110^th^ to 125^th^, 133^th^ to 150^th^, 164^th^ to 176^th^, and 178^th^ to 198^th^ residue and no *β*-strands. Figure S3. Homology models from different servers; (a) homology modelling by Swiss-Model server; (b) homology modelling by Phyre-2 server; and (c) Homology modelling by ConSurf; homology modelling by RaptorX server. Figure S4. Ramachandran plots of different models; (a) LEP_ Swiss-Model, (b) LEP_Phyre2, (c) LEP_ConSurf, and (d) LEP_RaptorX.

## References

[1] Schwartz M. W., Woods S. C., Porte D., Seeley R. J., Baskin D. G. (2000). Central nervous system control of food intake. *Nature*.

[2] Park H.-K., Ahima R. S. (2015). Physiology of leptin: energy homeostasis, neuroendocrine function and metabolism. *Metabolism*.

[3] Robertson S. A., Leinninger G. M., Myers M. G. (2008). Molecular and neural mediators of leptin action. *Physiology & Behavior*.

[4] Rodríguez V. M., Picó C., Portillo M. P., Macarulla M. T., Palou A. (2002). Dietary fat source regulates ob gene expression in white adipose tissue of rats under hyperphagic feeding. *British Journal of Nutrition*.

[5] Yang L., McKnight G. S. (2015). Hypothalamic PKA regulates leptin sensitivity and adiposity. *Nature Communications*.

[6] Myers M. G., Leibel R. L., Seeley R. J., Schwartz M. W. (2010). Obesity and leptin resistance: distinguishing cause from effect. *Trends in Endocrinology & Metabolism*.

[7] Zhou Y., Rui L. (2013). Leptin signaling and leptin resistance. *Frontiers of Medicine*.

[8] Ding C., Jin S. (2009). High-throughput methods for SNP genotyping. *Single Nucleotide Polymorphisms*.

[9] Wohlrab H. (2006). The human mitochondrial transport/carrier protein family. Nonsynonymous single nucleotide polymorphisms (nsSNPs) and mutations that lead to human diseases. *Biochimica et Biophysica Acta (BBA)-Bioenergetics*.

[10] Singh L. V., Jayakumar S., Sharma A. (2015). Comparative screening of single nucleotide polymorphisms in *β*-casein and *κ*-casein gene in different livestock breeds of India. *Meta Gene*.

[11] Mahrous K., Hassanane M., Shafey H., Mordy M. A., Rushdi H. (2016). Association between single nucleotide polymorphism in ovine Calpain gene and growth performance in three Egyptian sheep breeds. *Journal of Genetic Engineering and Biotechnology*.

[12] Rosenbloom K. R., Armstrong J., Barber G. P. (2015). The UCSC genome browser database: 2015 update. *Nucleic Acids Research*.

[13] Zerbino D. R., Achuthan P., Akanni W. (2018). Ensembl 2018. *Nucleic Acids Research*.

[14] Sim N.-L., Kumar P., Hu J., Henikoff S., Schneider G., Ng P. C. (2012). SIFT web server: predicting effects of amino acid substitutions on proteins. *Nucleic Acids Research*.

[15] Ng P. C., Henikoff S. (2003). SIFT: Predicting amino acid changes that affect protein function. *Nucleic Acids Research*.

[16] Bendl J., Stourac J., Salanda O. (2014). PredictSNP: robust and accurate consensus classifier for prediction of disease-related mutations. *PLoS Computational Biology*.

[17] Choi Y., Sims G. E., Murphy S., Miller J. R., Chan A. P. (2012). Predicting the functional effect of amino acid substitutions and indels. *PloS One*.

[18] Turkson J. (2004). STAT proteins as novel targets for cancer drug discovery. *Expert Opinion on Therapeutic Targets*.

[19] Hecht M., Bromberg Y., Rost B. (2015). Better prediction of functional effects for sequence variants. *BMC Genomics*.

[20] Ota N., Kurahashi R., Sano S., Takano K. (2018). The direction of protein evolution is destined by the stability. *Biochimie*.

[21] Tokuriki N., Stricher F., Serrano L., Tawfik D. S. (2008). How protein stability and new functions trade off. *PLoS Computational Biology*.

[22] Chen C.-W., Lin J., Chu Y.-W. (2013). iStable: off-the-shelf predictor integration for predicting protein stability changes. *BMC Bioinformatics*.

[23] Worth C. L., Preissner R., Blundell T. L. (2011). SDM—a server for predicting effects of mutations on protein stability and malfunction. *Nucleic Acids Research*.

[24] Rodrigues C. H., Pires D. E., Ascher D. B. (2018). DynaMut: predicting the impact of mutations on protein conformation, flexibility and stability. *Nucleic Acids Research*.

[25] Ashkenazy H., Abadi S., Martz E. (2016). ConSurf 2016: an improved methodology to estimate and visualize evolutionary conservation in macromolecules. *Nucleic Acids Research*.

[26] Ahmad H. I., Ijaz N., Afzal G. (2022). Computational Insights into the Structural and Functional Impacts of nsSNPs of bone morphogenetic proteins. *BioMed Research International*.

[27] Ahmad H. I., Afzal G., Iqbal M. N. (2021). Positive selection drives the adaptive evolution of mitochondrial antiviral signaling (MAVS) proteins-mediating innate immunity in mammals. *Frontiers in Veterinary Science*.

[28] Buchan D. W., Minneci F., Nugent T. C., Bryson K., Jones D. T. (2013). Scalable web services for the PSIPRED protein analysis workbench. *Nucleic Acids Research*.

[29] Jones D. T. (1999). Protein secondary structure prediction based on position-specific scoring matrices 1 1Edited by G. Von Heijne. *Journal of Molecular Biology*.

[30] Arnold K., Bordoli L., Kopp J., Schwede T. (2006). The SWISS-MODEL workspace: a web-based environment for protein structure homology modelling. *Bioinformatics*.

[31] Ahmad H. I., Afzal G., Sadia S. (2022). Structural and evolutionary adaptations of Nei-like DNA glycosylases proteins involved in base excision repair of oxidative DNA damage in vertebrates. *Oxidative Medicine and Cellular Longevity*.

[32] Biasini M., Bienert S., Waterhouse A. (2014). SWISS-MODEL: modelling protein tertiary and quaternary structure using evolutionary information. *Nucleic Acids Research*.

[33] Kelley L. A., Mezulis S., Yates C. M., Wass M. N., Sternberg M. J. (2015). The Phyre2 web portal for protein modeling, prediction and analysis. *Nature Protocols*.

[34] Ahmad H. I., Asif A. R., Ahmad M. J. (2020). Adaptive evolution of peptidoglycan recognition protein family regulates the innate signaling against microbial pathogens in vertebrates. *Microbial Pathogenesis*.

[35] Källberg M., Wang H., Wang S. (2012). Template-based protein structure modeling using the RaptorX web server. *Nature Protocols*.

[36] Lovell S. C., Davis I. W., Arendall W. B. (2003). Structure validation by C*α* geometry: *ϕ*, *ψ* and C*β* deviation. *Proteins: Structure, Function, and Bioinformatics*.

[37] Prince G. S. H., Dhar T. (2018). *A simulation analysis and screening of deleterious non-synonymous single nucleotide polymorphisms (SNPs) in human CDKN1A gene*.

[38] Geary T., McFadin E., MacNeil M. (2003). Leptin as a predictor of carcass composition in beef cattle. *Journal of Animal Science*.

[39] Bouafi H., Bencheikh S., Krami M. A. L. (2019). Prediction and structural comparison of deleterious coding nonsynonymous single nucleotide polymorphisms (nsSNPs) in human LEP gene associated with obesity. *Biomed Research International*.

[40] Nogueiras R., Tschöp M. H., Zigman J. M. (2008). Central nervous system regulation of energy metabolism: ghrelin versus leptin. *Annals of the New York Academy of Sciences*.

[41] Buchanan F. C., Fitzsimmons C. J., Van Kessel A. G., Thue T. D., Winkelman-Sim D. C., Schmutz S. M. (2002). Association of a missense mutation in the bovine leptin gene with carcass fat content and leptin mRNA levels. *Genetics Selection Evolution*.

[42] Yamada T., Kawakami S. I., Nakanishi N. (2003). The relationship between plasma leptin concentrations and the distribution of body fat in crossbred steers. *Animal Science Journal*.

[43] Kemp B. (2003). *Utilization of the cytosine to thymine missense mutation in the bovine leptin gene*.

[44] Hashemi A., Mardani K., Farhadian M., Ashrafi I., Ranjbari M. (2011). Allelic polymorphism of Makoei sheep leptin gene identified by polymerase chain reaction and single strand conformation polymorphism. *African Journal of Biotechnology*.

[45] Zhou H., Hickford J. G., Gong H. (2009). Identification of allelic polymorphism in the ovine leptin gene. *Molecular Biotechnology*.

[46] Marie M., Findlay P., Thomas L., Adam C. L. (2001). Daily patterns of plasma leptin in sheep: effects of photoperiod and food intake. *Journal of Endocrinology*.

[47] Hajihosseinlo A., Hashemi A., Sadeghi S. (2012). Association between polymorphism in exon 3 of leptin gene and growth traits in the Makooei sheep of Iran. *Livestock Research for Rural Development*.

[48] Shojaei M., Mohammad Abadi M., Asadi Fozi M., Dayani O., Khezri A., Akhondi M. (2011). Association of growth trait and Leptin gene polymorphism in Kermani sheep. *Journal of Cell and Molecular Research*.

[49] Barzehkar R., Salehi A., Mahjoubi F. (2009). Polymorphisms of the ovine leptin gene and its association with growth and carcass traits in three Iranian sheep breeds. *Iranian Journal of Biotechnology*.

[50] Boucher D., Palin M., Castonguay F., Gariépy C., Pothier F. (2006). Detection of polymorphisms in the ovine leptin (LEP) gene: association of a single nucleotide polymorphism with muscle growth and meat quality traits. *Canadian Journal of Animal Science*.

[51] Vignal A., Milan D., SanCristobal M., Eggen A. (2002). A review on SNP and other types of molecular markers and their use in animal genetics. *Genetics Selection Evolution*.

[52] Solayman M., Saleh M. A., Paul S., Khalil M. I., Gan S. H. (2017). In silico analysis of nonsynonymous single nucleotide polymorphisms of the human adiponectin receptor 2 (ADIPOR2) gene. *Computational Biology and Chemistry*.

[53] Patel J. B., Chauhan J. B. (2018). Computational analysis of non-synonymous single nucleotide polymorphism in the bovine cattle kappa-casein (CSN3) gene. *Meta Gene*.

[54] Anfinsen C. B. (1973). Principles that govern the folding of protein chains. *Science*.

[55] Ahmad H. I., Zhou J., Ahmad M. J. (2020). Adaptive selection in the evolution of programmed cell death-1 and its ligands in vertebrates. *Aging (Albany NY)*.

[56] Araya C. L., Fowler D. M., Chen W., Muniez I., Kelly J. W., Fields S. (2012). A fundamental protein property, thermodynamic stability, revealed solely from large-scale measurements of protein function. *Proceedings of the National Academy of Sciences*.

[57] Bloom J. D., Labthavikul S. T., Otey C. R., Arnold F. H. (2006). Protein stability promotes evolvability. *Proceedings of the National Academy of Sciences*.

[58] Bloom J. D., Raval A., Wilke C. O. (2007). Thermodynamics of neutral protein evolution. *Genetics*.

[59] Bershtein S., Goldin K., Tawfik D. S. (2008). Intense neutral drifts yield robust and evolvable consensus proteins. *Journal of Molecular Biology*.

