# Supplementary material for: A Simulation Analysis and Screening of Deleterious Nonsynonymous Single Nucleotide Polymorphisms (nsSNPs) in Sheep LEP Gene
Source: Biomed Res Int. 2022 Aug 8;2022:7736485. doi: 10.1155/2022/7736485 (PMC9377880; doi:10.1155/2022/7736485)
Supplement: Supplementary Materials — Table S1. Deleterious SNPs predicted by predictSNP2. Table S2. nsSNP analysis by SNAP2. Table S 3. nsSNP analysis by PROVEAN. Table S4. SDM and DynaMut tools analysis results of the effect of missense mutations on protein stability. Figure S1. Prediction of evolutionary conserved amino acid residues by ConSurf server. Conservation score is represented as the color coding bars. Figure S2. Protein secondary structure predictions by PSIPRED tool. The graphical output of PSIPRED prediction of secondary structure of the sheep LEP protein shows 6 α-helices extends from 41th to 55th, 66thto 84th, 110th to 125th, 133th to 150th, 164th to 176th, and 178th to 198th residue and no β-strands. Figure S3. Homology models from different servers; (a) homology modelling by Swiss-Model server; (b) homology modelling by Phyre-2 server; and (c) Homology modelling by ConSurf; homology modelling by RaptorX server. Figure S4. Ramachandran plots of different models; (a) LEP_ Swiss-Model, (b) LEP_Phyre2, (c) LEP_ConSurf, and (d) LEP_RaptorX. [file 7736485.f1.docx]

**A Simulation Analysis and Screening of Deleterious Non-Synonymous Single Nucleotide Polymorphisms (nsSNPs) in Sheep *LEP* Gene**

**Shishay Girmay^1,*^ Hafiz Ishfaq Ahmad^2^_,_ and**  **Quratul Ain Zahra^3^**

^1^Department of Animal Science, College of Dryland Agriculture, Samara University, Ethiopia

^2^Department of Animal Breeding and Genetics, University of Veterinary and Animal Sciences, Ravi Campus, Pattoki, Pakistan. [ishfaq.ahmad@uvas.edu.pk](mailto:ishfaq.ahmad@uvas.edu.pk)

^3^Woman medical officer DHQ hospital rajanpur ,punjab Pakistan [quratulainzahra313@gmail.com](mailto:quratulainzahra313@gmail.com)

*Correspondence Should be addressed to Shishay Girmay; [shishaygirmay@su.edu.et](mailto:shishaygirmay@su.edu.et)

**Table S1.** Deleterious SNPs predicted by predictSNP2

| Mutation | Prediction tools | | | | | | |
| --- | --- | --- | --- | --- | --- | --- | --- |
|  | predictSNP | MAPP | PhD-SNP | PolyPhen-1 | Polyphen-2 | SNAP | PANTHER |
| R2C | Deleterious | - | Neutral | Deleterious | Deleterious | Deleterious | Deleterious |
| L35P | Neutral | - | Neutral | Neutral | Neutral | Deleterious | Deleterious |
| T75M | Deleterious | Deleterious | Neutral | Deleterious | Deleterious | Deleterious | Deleterious |
| T86M | Deleterious | Deleterious | Neutral | Deleterious | Deleterious | Deleterious | Deleterious |
| V94I | Deleterious | Deleterious | Neutral | Neutral | Neutral | Neutral | Neutral |
| D98N | Deleterious | Deleterious | Neutral | Deleterious | Deleterious | Deleterious | Deleterious |
| N136T | Deleterious | Deleterious | Deleterious | Deleterious | Deleterious | Deleterious | Neutral |
| R142Q | Neutral | Neutral | Neutral | Neutral | Deleterious | Neutral | Deleterious |
| P157Q | Deleterious | Deleterious | Neutral | Deleterious | Deleterious | Neutral | Neutral |
| V181L | Deleterious | Deleterious | Neutral | Deleterious | Deleterious | Deleterious | Neutral |
| R196Q | Neutral | Neutral | Neutral | Neutral | Neutral | Neutral | Neutral |

Prediction of LEP nsSNPs using SNAP2 showed that five nsSNPs (L35P, T75M, V94I, D98N, and V181L) have a damaging eﬀect with scores ranging from 7 to 78. The results of the SNP2 are presented in **Table S2.**

**Table S2. nsSNP analysis by SNAP^2^**

| nsSNPs | Substitution | Predicted eﬀect | Score | Expected accuracy |
| --- | --- | --- | --- | --- |
| rs587813135 | R2C | Neutral | -9 | 53% |
| rs414488761 | L35P | Effect | 51 | 75% |
| rs1086818376 | T75M | Effect | 78 | 85% |
| rs593507294 | T86M | Neutral | -99 | 97% |
| rs592349134 | V94I | Effect | 7 | 53% |
| rs426762318 | D98N | Effect | 72 | 85% |
| rs429690456 | N136T | Neutral | -1 | 53% |
| rs409584889 | R142Q | Neutral | -3 | 53% |
| rs1093355763 | P157Q | Neutral | -6 | 53% |
| rs420693815 | V181L | Effect | 42 | 71% |
| rs428185456 | R196Q | Neutral | -54 | 72% |

All the nsSNPs predicted as a deleterious and damaging by SIFT, predictSNP2 and SNAP2 were further reconﬁrmed by PROVEAN tool. The PROVEAN tool characterises functional amino acid substitution through evolutionary relationships classiﬁcation in LEP protein. Through employing, the PROVEAN server, 4 out of 11 nsSNPs were verified deleterious, T75M (PROVEAN score −4.876), D98N (PROVEAN score −4.274), N136T (PROVEAN score −3.989) and P157Q (PROVEAN score −2.680). The results of PROVEAN are present in **Table S3.**

**Table S 3.** nsSNP analysis by PROVEAN

| Variant | PROVEAN score | Prediction (cut off= -2.5) |
| --- | --- | --- |
| R2C | 0.000 | Neutral |
| L35P | 0.000 | Neutral |
| T75M | -4.876 | Deleterious |
| T86M | 0.516 | Neutral |
| V94I | -0.157 | Neutral |
| D98N | -0.4274 | Deleterious |
| N136T | -3.989 | Deleterious |
| R142Q | -0.871 | Neutral |
| P157Q | -2.680 | Deleterious |
| V181L | -2.059 | Neutral |
| R196Q | 0.407 | Neutral |

**Table S4.** SDM and DynaMut tools analysis results of the effect of missense mutations on protein stability.

|  | SDM | | DynaMut | | | |
| --- | --- | --- | --- | --- | --- | --- |
| Variant | ΔΔG | Outcome | ΔΔG (kcal/mol) | Outcome | ΔΔS_Vib_ ENCoM  (kcal.mol-1.K-1) | Outcome |
| T75M | -1.24 | Reduced stability | 0.587 | Stabilising | -0.027 | Decrease of molecular flexibility |
| T86M | 0.09 | Increased stability | 0.030 | Stabilising | -0.046 | Decrease of molecular flexibility |
| V94I | -0.12 | Reduced stability | 0.742 | Stabilising | -0.272 | Decrease of molecular flexibility |
| D98N | 0.07 | Increased stability | 0.269 | Stabilising | -0.056 | Decrease of molecular flexibility |
| N136T | -0.16 | Reduced stability | -0.035 | Destabilising | 0.041 | Increase of molecule flexibility |
| R142Q | -2.15 | Reduced stability | -0.907 | Destabilising | 0.441 | Increase of molecule flexibility |
| P157Q | 0.80 | Increased stability | 0.742 | Stabilising | -0.027 | Decrease of molecular flexibility |
| V181L | -2.92 | Reduced stability | 0.499 | Stabilising | -0.304 | Decrease of molecular flexibility |


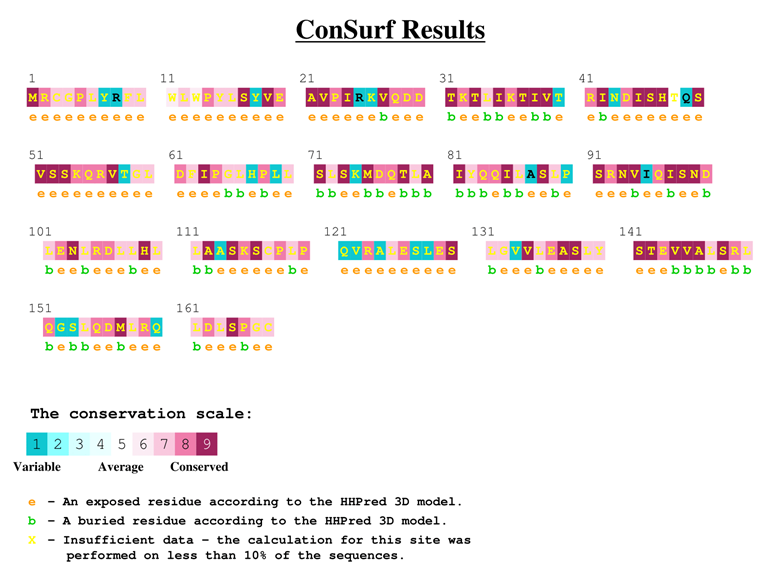


**Figure S1.** Prediction of evolutionary conserved amino acid residues by ConSurf server. Conservation score is represented as the color coding bars.


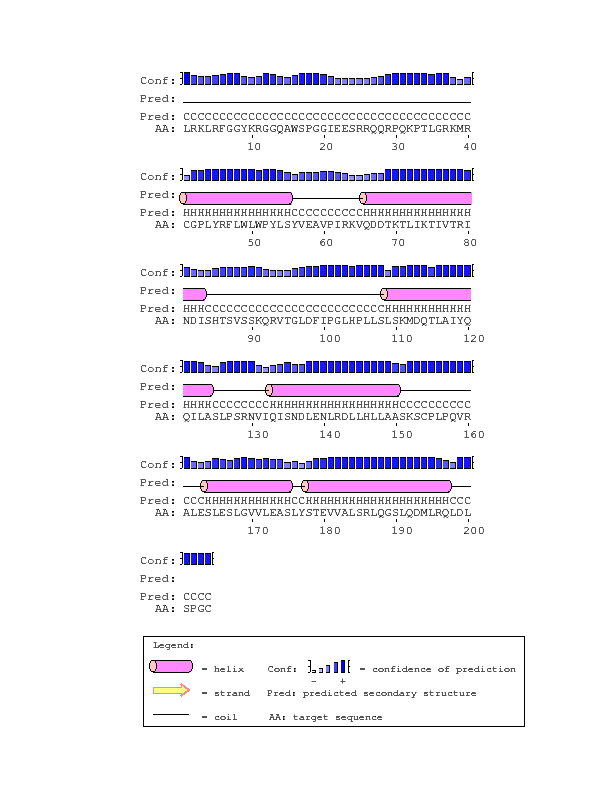


**Figure S2.** Protein secondary structure predictions by PSIPRED tool. The graphical output of PSIPRED prediction of secondary structure of the sheep LEP protein shows 6 α-helices extends from 41^th^ to 55^th^, 66^th to^ 84^th^, 110^th^ to 125^th^, 133^th^ to 150^th^, 164^th^ to 176^th^, and 178^th^ to 198^th^ residue and no β-strands.


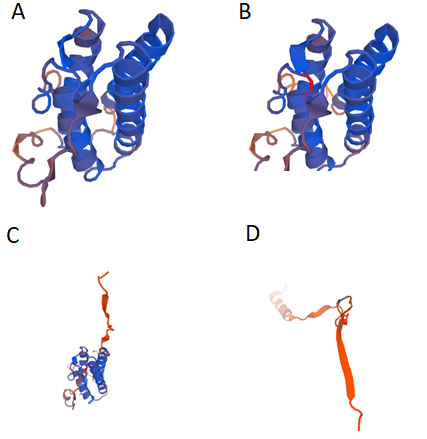


**Figure S3.** Homology models fom different servers; A. Homology modelling by Swiss-Model server; B. Homology modelling by Phyre-2 server, C. Homology modelling by ConSurf; Homology modelling by RaptorX server


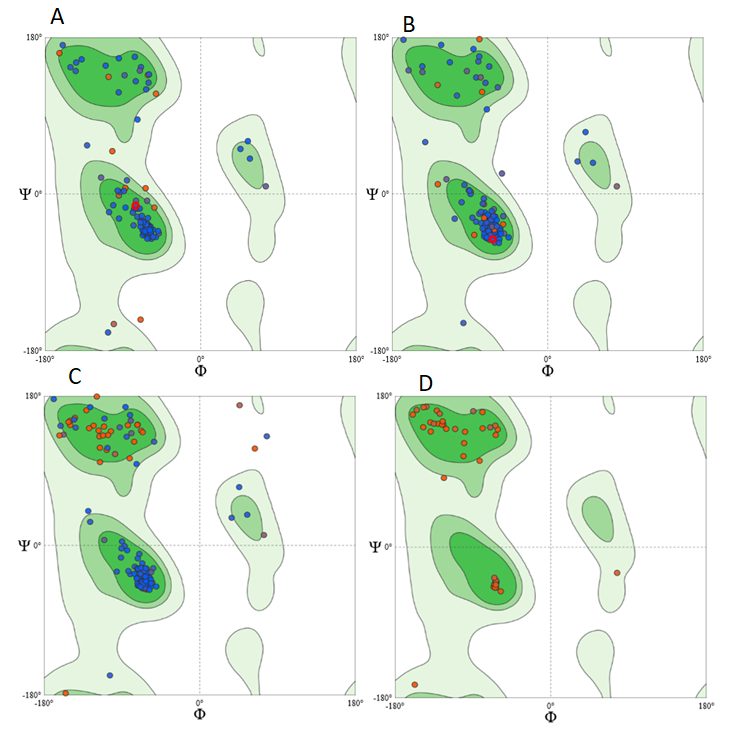


**Figure S4.** Ramachandran plots of different models; A. LEP_ Swiss model, B. LEP_Phyre2, C. LEP_ConSurf, D. LEP_RaptorX
